# Pediatric Quality of Life Inventory^TM^ version 4.0 short form generic core scale across pediatric populations review data

**DOI:** 10.1016/j.dib.2021.107599

**Published:** 2021-11-24

**Authors:** Matthew Smyth, Kevan Jacobson

**Affiliations:** a Department of Pediatrics, Division of Gastroenterology, Hepatology and Nutrition, Faculty of Medicine, British Columbia Children's Hospital, University of British Columbia, British Columbia, Canada; b British Columbia Children Hospital Research Institute, Vancouver, British Columbia, Canada; c Department of Cellular and Physiological Sciences, University of British Columbia, Vancouver, British Columbia, Canada

**Keywords:** Quality of life, Inflammatory bowel disease, Pediatrics, PedsQL^TM^

## Abstract

The Pediatric Quality of Life Inventory^TM^ Version 4.0 Short Form Generic Core Scale (PedsQL^TM^) is a validated and widely used tool assessing the quality of life (QoL) of children and youth. It has been used extensively across healthy populations as well as those with chronic and acute illnesses, allowing for comparison of the psychosocial impact of chronic illness between pediatric disease cohorts. As part of the QoL initiative undertaken at the British Columbia Children's Hospital (BCCH) Inflammatory Bowel Disease (IBD) program and published in the Journal of Pediatrics titled “Cross-Sectional Analysis of Quality of Life in Pediatric Patients with IBD in British Columbia, Canada,” a limited literature review was conducted using Embasse and Ovid. Studies using the English version of the PedsQL^TM^ short form generic scale (not a disease specific scale) were identified. Studies with populations greater than 50 patients with robust subgroup sample size were included, with an emphasis on studies with well-defined patients with chronic disease. These data were compared to the BCCH population, as discussed in the aforementioned journal article.

Analysis within the BCCH cohort is described separately. Comparison between different populations from the existing literature was qualitative only, with no statistical analysis done given the heterogeneity of populations and studies.

In a study of patients from the emergency department at BCCH (*n=*178), the mean (SD) QoL scores of the healthy patients was 89.2 (10.3). In a group of self-identified healthy patients in California (*n=*5079), their mean QoL score was 83.9 (12.5). Separating the BCCH IBD population by disease activity, those in remission (*n=*220, 84.4 (12.8)) have similar QoL scores to these healthy cohorts, though their scores remain slightly below the previously published BCCH cohort. For children with any degree of active IBD (*n=*98, 75.6 (15.8)), their QoL scores are below the healthy means and are lower than other groups with self-identified “chronic illnesses” (*n=*367, 77.2 (15.5)), diabetes (*n=*418, 82.3 (13.5)), mild asthma (*n=*281, 85.5 (13.3)), or Canadian patients 4 weeks post-concussion (*n=*1157, 80.3). BCCH IBD patients with moderately to severely active disease have QoL scores well below the other disease groups (*n=*33, 63.1 (18.8)); lower than oncology patients on induction chemotherapy regimens (*n=*105, 68.9 (16.0)), acute inpatients (*n=*359, 63.9 (20.3)), and asthmatics with moderate-severe, persistent asthma (*n=*86, 67.1 (18.6)).

This data is useful for clinicians treating pediatric patients looking at how QoL is influenced by chronic illness and by factors such as disease type and severity.

## Specifications Table

**Table:** 

Subject	Perinatology, Paediatrics and Child Health
Specific subject area	Quality of Life in Paediatric patients with chronic illness
Type of data	Table
How data were acquired	REDCap survey of PedsQL^TM^ (noted included because of third party copyright); Embasse and OVID literature search;
Data format	Analyzed Filtered Raw
Parameters for data collection	Patient age, diagnosis and disease activity collected as part of BCCH IBD dataset. EMBASS and OVID articles identified that used the English version of the PedsQL^TM^ short form generic scale. Those with total populations greater than 50 patients with robust subgroup population sizes were included, with an emphasis on studies with well-defined in patients with chronic illness.
Description of data collection	BCCH IBD Data collected as part of Quality Improvement initiative using an iPad based REDCap survey (that included the PedsQL^TM^ generic tool) at the time of clinical encounter. Literature search conducted using Embass and OVID.
Data source location	Institution: British Columbia Children's Hospital City/Town/Region: Vancouver Country: Canada
Data accessibility	Repository name: Mendeley Data identification number: http://dx.doi.org/10.17632/r7fyyf9fhc.1 Direct URL to data: http://dx.doi.org/10.17632/r7fyyf9fhc.1
Related research article	M. Smyth, J. Chan, K. Evans, C. Penner, A. Lakhani, T. Newlove, K. Jacobson, Cross-Sectional Analysis of Quality of Life in Pediatric Patients with Inflammatory Bowel Disease in British Columbia, Canada., Pediatrics, In Press.

## Value of the Data

• To provide a comparison of quality of life between various pediatric chronic illnesses as well as their healthy peers using a validated, generic, and widely used quality of life measurement tool.
• This information is useful for clinicians caring for pediatric patients with a variety of chronic illnesses.
• As the PedsQL^TM^ continues to be widely used in clinical care and research alike, this data will continue to be useful in providing a benchmark for results across patient populations. By breaking down results by both disease type and disease severity/disease activity, and ensuring conclusions are drawn with robust population sizes, this data should provide the standard by which future PedsQL^TM^ research is conducted.
• This data enables the reader to appreciate the psychosocial burden of disease across populations and anticipate the needs of the patient. By understanding the quality of life implications of a chronic diagnosis, the hope is that the practitioner can work with families and community supports to help children and youth thrive despite their diagnosis.

## 1 Data Description

Table 1: this table corresponds to supplemental Table 3 from the article “Cross-Sectional Analysis of Quality of Life in Pediatric Patients with Inflammatory Bowel Disease in British Columbia, Canada” published in the Journal of Pediatrics.

**Table 1:** looks at the published literature of healthy controls and chronic conditions where patients have used the self-reported PedsQL 4.0 Generic or Short form questionnaire. This table corresponds to Table 3 from the article by M.Smyth et al. “Cross-Sectional Analysis of Quality of Life in Pediatric Patients with Inflammatory Bowel Disease in British Columbia, Canada” in press with the Journal of Pediatrics.

Study	Comment	Subgroup	N	QOL score	SD
**BCCH IBD Patients**		Total (a)	351	79.95	15.77
		Remission (b)	220	84.41	12.84
		Mild (c)	98	75.59	15.75
		Moderate-severe (d)	33	63.13	18.78

**Health Controls**					

Kruse et al. [1]	**BCCH Population**. Healthy patients discharged from emerg, age 8-16. ∧SD derived from reported confidence interval	Healthy	178	89.17	10.28∧
Varni et al [2]	California pediatric population responding to mail out PedsQL; only healthy population included	Healthy	5079	83.91	12.47
Williams et al. [3]	Australia: 9-12 year old healthy as part of obesity survey	Healthy	1099	80.5	12.2
Varni et al [4]	California: Phone survey from healthy patients identified in ortho clinic as "recovered"	Healthy	401	83	14.79
Dierderen et al. [5]	Netherlands: Age 8-18; Online questionnaire self-identified as healthy ∧Median, IQR	Healthy	340	83.15∧	77.17-90.22
Youssef et al. [6]	New Jersey: Healthy patients seen for routine appointment or minor acute medical problem; prospectively enrolled	Healthy	42	87.7	14.7
Tahirovic et al. [7]	Bosnia and Herzegovina: Healthy visitors to pediatrics department, no chronic conditions ∧pooled mean + SD for age 8-18	Healthy	71	88.77∧	16.76∧

**Chronically Ill Pediatric Populations**					

Novak et al. [8]	Concussion Study: Canadian patients; 9 Centres, 8-18 yo	All Patients post concussion	1157	80.3	Not reported
		4 wks post with persisting Sx's	510	70	Not reported
Young et al. [9]	Hemophilia study: Toronto Boys 6-17 yo, prospectively enrolled	Hemophilia	60	80.9	13.69
Chan et al. [10]	Asthma Study: Asthma severity based off NHLBI Guidelines; 13 pediatric sites across US	Mild intermittent asthma	281	85.4	13.3
		Mild persistent asthma	96	75	15.2
		moderate-severe persistent asthma	86	67.1	18.6
Varni et al. [4]	Cancer Study in California: 8-18 yo; includes inpatients/outpatiens, all cancer types, including remission and recurrent dx	Cancer- On Tx	105	68.92	15.97
		Cancer- Off Tx < 12Mo	41	70.88	17.19
		Cancer- Off Tx >12 Mo	73	77.66	15.25
Varni et al. [11]	Type 1 Diabetes: 13-25 yo across 10 american sites, those with poorly controlled DM have lower QoL scores	T1DM	418	82.33	13.53
Desai et al. [12]	Inpatient population age 13-18 admitted to Seattle Childrens with a varient of Dx. Prospectively enrolled	Inpatient	359	63.9	20.28
Varni et al. [13]	Rheumatologic Dx in California: JIA, fibromyalgia, spondyloarthritis, SLE, Other (157) Ages 6-18	Rheumatological illnesses	336	70.35	17.83
Goldstein et al. [14]	End Stage Renal Disease from two american centres, ages 5-18	ESRD, including dialysis and transplant patients	85	73.97	15.22
Tahirovic et al. [7]	Congintal Heart Disease in Bosnia and Herzegovina: Patients 1+ years post cardiac surgery for CHD ∧pooled mean + SD for age 8-18	Congenital Heart Disease	83	87.35∧	12.47∧
Ng et al. [15]	Liver Transplant study in Canada + US, patients 10 years post LTx; retrospective. Mean age of LTx 2.3yo	Liver Transplant patients	73	77.16	12.93
Maskell et al. [16]	Burn patients; Australia and NZ: Age 8-17; not acute burns, with mature scarring present; 6 sites	Burns	66	78.87	15.1
Younossi et al. [17]	Chronic HCV patients receiving sofosbuvir and ribavirin; Prospective, International (30 sites, 7 countries)	Hepatitis C Virus (HCV)	50	80.4	1.93
Liu et al. [18]	Inflammatory Brain Diseases: QoL scores from time of Dx; most common presenting sx's: seizures, cognitive dysfunction or hemiparesis. International, multi-centre	Inflammatory Brain Diseases	34	68.4	Not reported

**Chronically Ill- Self Identified**					

Varni et al. [8]	Surveys completed in community specialty clinics in United States; Patients self-identified as chronically. Those in subspecialty clinics identified as acutely ill	Chronically Ill	367	77.19	15.53
		Acutely Ill	148	78.7	14.03
Varni et al. [2]	Surveys from 18 elementary, 4 middle, and 3 high schools in California; Parents identified child as chronically ill	Chronically Ill	100	71.59	16.17

**Overweight and Obese**					

Williams et al. [3]	Overweight/Obese 9-12 year olds; Ht and Wt measured at schools by trained staff; categories based off international Obesity task force; Australia	Overweight	294	79.3	12.8
		Obese	63	74	14.2
Hoedjes et al. [19]	Severe Obesity (SDS-BMI >3, or >2.3 with obesity-related comorbidity); Prospective, Netherlands ∧SD derived from SE.	Severe Obsity	120	67.8	∧19.7
Faus et al. [20]	BMI >85% for age; Convenience Sample. New Jersey	Obese	60	76.42	Not reported

**Gastrointestinal Illness**					

Varni et al. [21]	GI disorders; 9 US centres across US ages 5-18	Functional GI disorders (constipation, Pain, IBS, dyspepsia)	281	70.2	17
		Organic GI Disorders (IBD and GERD)	298	78	14.6
Varni et al. [22]	Outpatient GI population in 3 US sites; 2002-2004 ages 5-18	IBS (Rome Criteria)	119	77.9	12.64
		Functional Abdominal Pain (Rome)	81	79.98	10.62
Youssef et al.[6]	Single NJ Centre, prospective, 5-18 yo. (Chronic constipation >3 months sx's with <3 BMs/week)	Chronic Constipation	80	70.4	12.2
		IBD, New diagnosis	42	83.8	13.2
		GERD (Bx proven w/sx's)	56	79.9	14
Kunz et al [23]	IBD patients recruited from 3 american sites	IBD- Remission	79	86.67	13.31
		IBD- Mild-Severe	42	78.57	17.99
Faus et al. [20]	IBD pts: 80% remission, 20% mild; Convenience Sample; NJ, USA	IBD	60	79.3	Not reported
Dierderen et al. [5]	IBD pt's: 63% patients in remission; Cross-sectional study of online questionnaires in Netherlands. ∧ Median and IQR	IBD	87	83.37∧	71.5-91.3∧

BCCH: BC Children's Hospital; BMI: Body Mass Index; Dx: diganosis; GI: Gastrointestinal HCV: Heptatitis C Virus; IBD: Inflammatory Bowel Disease; IQR: Interquartile Range; JIA: jeuvenile idiopathic arthrtitis; LTx: liver transplant; NHLBI: National Heart Lung and Blood Institute; NJ: New Jersey; NZ: New Zealand; QOL: Quality of Life; SD: Standard Deviation; SLE: systemic lupus erythematosus.

The table shows the results from the pediatric Inflammatory Bowel Disease (IBD) population at BC Children's Hospital (BCCH) as well as the PedsQL^TM^ scores from multiple other large studies that used the Quality of Life (QoL) tool. In a study of patients from the emergency department at BCCH (*n=*178), the mean (SD) QoL scores of the healthy patients was 89.2 (10.3). In a group of self-identified healthy patients in California (*n=*5079), their mean QoL score was 83.9 (12.5). Separating the BCCH IBD population by disease activity, those in remission (*n=*220, 84.4 (12.8)) are similar to these healthy scores, though remain slightly below the previously published BCCH cohort. For children with any degree of active IBD (*n=*98, 75.6 (15.8)), their QoL scores are below the healthy means and are lower than other groups with self-identified “chronic illnesses” (*n=*367, 77.2 (15.5)), diabetes (*n=*418, 82.3 (13.5)), mild asthma (*n=*281, 85.5 (13.3)), or Canadian patients 4 weeks post-concussion (*n=*1157, 80.3). BCCH IBD patients with moderately to severely active disease have QoL scores well below the other disease groups (*n=*33, 63.1 (18.8)); lower than oncology patients on induction chemotherapy regimens (*n=*105, 68.9 (16.0)), acute inpatients (*n=*359, 63.9 (20.3)), and asthmatics with moderate-severe, persistent asthma (*n=*86, 67.1 (18.6)). The raw data for this table is attached and is also available on an open data repository.

Data Upload: The raw data is uploaded in both .csv and .sav format

Supplemental Data: Original Article: The original research article, in press with the Journal of Pediatrics, is attached.

PedsQL^TM^ Pediatric Quality of Life Inventory Version 4.0 Short Form: The short form of the survey was used to collect the quality of life data for this study. The authors do not own the rights to this tool, and so will provide a summary of the tool only. The tool uses a standard 5 point Likert scale for patients to respond to each question. There are four sections to the short form questionnaire, with 3-5 questions per section for a total of 15 questions. The first section looks at any issues being able to do normal activities of childhood and participating with peers; the second section looks at frequency of low mood symptoms; the third section asks about interpersonal difficulties with peers; the fourth section looks at difficulty with classwork specifically.

## 2 Experimental Design, Materials and Methods

BCCH IBD QoL Data:

This data is from is a cross-sectional, retrospective study analyzing a quality improvement initiative in the IBD program at British Columbia Children's Hospital (BCCH), Vancouver, Canada. From 2014-2018, a multidisciplinary team of pediatric gastroenterologists, IBD nurses, and clinical psychologists at BCCH started a program to identify and support IBD patients with psychosocial issues associated with their disease. QoL was assessed using the Pediatric Quality of Life Inventory^TM^ Version 4.0 Short Form Generic Core Scale (PedsQL^TM^) [24], accessed via the hospital's licence. This tool was selected for its brevity and lack of questions overlapping with specific IBD symptoms, its validation in our target age group, and its straightforward scoring based off a Likert scale. The PedsQL^TM^ was programed into a REDCap [25] survey accessed on iPads donated by the BCCH Foundation. The disease activity at the time of survey completion was determined as part of clinical care, and patients were separated into disease activity categories that included remission, mild and moderate/severe disease. The overall QoL scores of the patient cohort as well as the QoL scores by disease activity are presented in the table (mean with standard deviation).

QoL scores from other patient populations:

After identifying appropriate studies (those with large, well-defined cohorts of patients with chronic illnesses and healthy controls) from EMBASS and OVID since 2003 (time of PedsQL^TM^ publication), mean QoL scores from the PedsQL^TM^ were extracted, along with standard deviations, where possible. For some studies [5], IQR was given and is presented, and for other studies [1,7,19], the SD was derived from the data in the manuscript and a standard deviation is presented.

## Ethics Statement

Ethical Considerations: This study evaluates a quality improvement initiative, and after consultation with the BCCH Research Ethics Board and in accordance with National TCPS2 policy, the study did not require an official ethics review.

## CRediT authorship contribution statement

**Matthew Smyth:** Conceptualization, Methodology, Formal analysis, Data curation, Writing – original draft, Visualization. **Kevan Jacobson:** Conceptualization, Methodology, Validation, Writing – review & editing, Visualization, Supervision.

## Declaration of Competing Interest

The authors declare that they have no known competing financial interests or personal relationships which have or could be perceived to have influenced the work reported in this article.

Outside of this submitted work, KJ is a Senior Clinician Scientists supported by the BC Children's Hospital Research Institute Clinician Scientist Awards Program and the Children with Intestinal and Liver Disorders (CHILD) Foundation. He has received research support from Janssen, AbbVie and adMare Bioinnovations, Vancouver, BC Canada. KJ has served on the advisory boards of Janssen, AbbVie, Merck and Mylan Inc and has participated in a speaker's bureau for Abbvie and Janssen.

## Appendix Supplementary materials

Image, application 1

Image, application 2
